# Fully customized placement of orthodontic miniplates: a novel clinical technique

**DOI:** 10.1186/1746-160X-10-14

**Published:** 2014-05-03

**Authors:** Jan Hourfar, Georgios Kanavakis, Peter Goellner, Björn Ludwig

**Affiliations:** 1 Department of Orthodontics, University of Heidelberg, Heidelberg, Germany; 2 Department of Orthodontics and Dentofacial Orthopedics, Tufts University School of Dental Medicine, 1 Kneeland Street DHS#1145, Boston, MA, USA; 3 Private practice, Bern, Switzerland; 4 Department of Orthodontics, University of Saarland, Homburg/Saar, Germany

## Abstract

**Introduction:**

The initial stability and survival rate of orthodontic mini-implants are highly dependent on the amount of cortical bone at their insertion site. In areas with limited bone availability, mini-plates are preferred to provide effective skeletal anchorage. The purpose of this paper was to present a new clinical technique for the insertion of mini-plates.

**Methods:**

In order to apply this new technique, a cone-beam image of the insertion area is required. A software (Galaxy Sirona, Bensheim, Germany) is used to construct a three-dimensional image of the scanned area and to virtually determine the exact location of the mini-plate as well as the position of the fixation screws. A stereolithographic model (STL) is then created by means of a three-dimensional scanner.

Prior to its surgical insertion, the bone plate is adapted to the stereo-lithographic model. Finally, a custom transfer jig is fabricated in order to assist with accurate placement of the mini-plate intra-operatively.

**Results:**

The presented technique minimizes intra-operative decision making, because the final position of the bone plate is determined pre-surgically. This significantly reduces the duration of the surgical procedure and improves its outcome.

**Conclusions:**

A novel method for surgical placement of orthodontic mini-plates is presented. The technique facilitates accurate adaptation of mini-plates and insertion of retaining surgical screws; thereby enabling clinicians to more confidently increase the use of bone plates, especially in anatomical areas where the success of non-osseointegrated mini-screws is less favorable.

## Introduction

Orthodontic mini-implants (MIs) have gained popularity among orthodontists mainly because they provide an effective tool in orthodontic cases with high anchorage demands. However, there are several factors affecting the survival rate of the implants that need to be taken into consideration prior to their insertion [1]. Previous literature has emphasized the significance of cortical bone thickness for initial stability of orthodontic mini-implants [2-4].

Clinical investigations assessing quality and quantity of alveolar bone in the maxilla and the mandible revealed that there are not many areas of sufficient bone quality able to guarantee successful placement of MIs [5-9].

A particularly challenging area is the anterior mandible. For cases that require unilateral or bilateral protraction of mandibular posterior teeth, placement of a skeletal anchorage device around the canine area can provide suitable possibilities supporting treatment mechanics. However, the only inter-radicular spaces in the mandible presenting adequate bone quality and quantity are distal to the first premolars [9]. Moreover, insertion of mini-implants in the mandibular canine region is not recommended in patients younger than 11 years of age, because of incomplete bone maturation and due to increased risk of interrupting normal eruption of the permanent canine [10,11].

In order to overcome the above-mentioned limiting factors, orthodontic mini-plates can be recommended as anchorage devices. The introduction of mini-plates in orthodontics has further enhanced treatment possibilities for complex orthodontic and orthopedic problems [12]. There are numerous reports in the literature proposing the use of mini-plates to address various orthodontic treatment needs, such as molar intrusion [13-15], correction of anterior open bite [16-18], maxillary [19,20] and mandibular molar [21] distalization, maxillary protraction [22-24] and maxillary impaction [25].

Mini-plates have a very high success rate (91.4% - 100%), low morbidity and are usually well accepted by patients [26]. In addition, there are few restrictions regarding the insertion sites for mini-plates; their placement is less dependant on the anatomy of the mucogingival tissues [27]. In contrast to mini-implants, the fixation screws of bone plates are mostly placed sub-apically, where bone quality is adequate. Thereby, tooth movements are also performed without obstruction and the risk of injuring the roots of adjacent teeth, during placement, is eliminated [11,28,29]. It has also been suggested that mini-plates may provide more reliable anchorage when higher forces, such as orthopedic forces, are needed [22,23].

Despite the efficiency of utilizing mini-plates as skeletal anchorage, there is a major drawback compared to mini-implants, i.e. the need for a surgical procedure to secure the plates with titanium screws. For placement in the anterior mandible, it is essential to adapt the mini-plates to the bony contour prior to fixation [27]. This process is not only technique sensitive, but can also be time consuming. Therefore, mini-plates are not as commonly used as mini-implants, although they present a high acceptance rate among both, orthodontists and patients [30,31].

Taking into consideration the disadvantages associated with the placement of orthodontic mini-plates, a new technique is presented to help simplify the process, reduce the time of the surgery and possible decrease failure rates. The technique allows for pre-surgical adaption of the mini-plates to the bony contour of a printed model of the insertion area and utilizes a custom made transfer jig to accurately position the plates during surgery.

## Materials and methods

**Figure 1:**
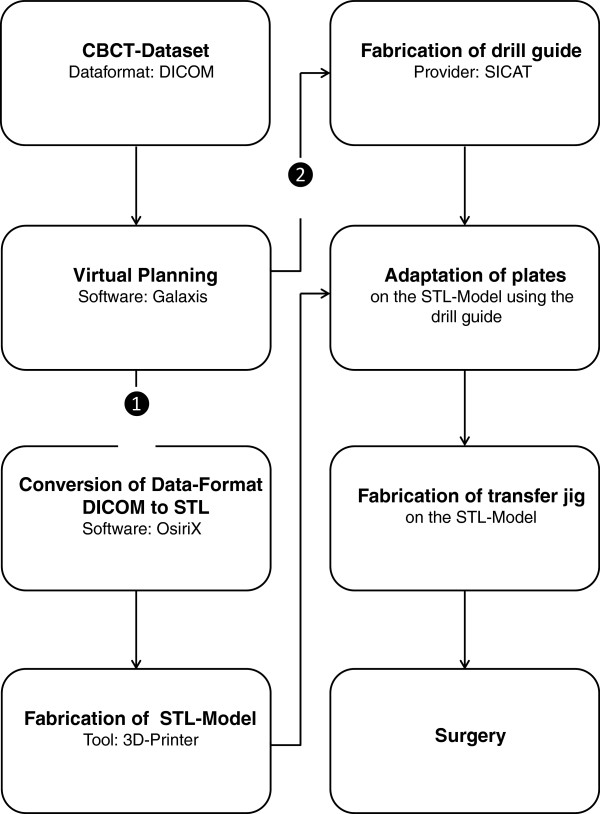
Diagrammatic sequence of steps for the proposed technique.

The proposed technique begins with virtual planning of the surgical procedure and the creation of a stereo-lithographic model as well as a drill guide. The orthodontic mini-plates are then adapted with the aid of the drill guide to the model and a transfer jig is fabricated. The jig is subsequently used to accurately position the plates during the surgical procedure. A schematic overview of all stages of the technique is displayed in Figure 1.

The present article is not an experimental clinical study. Patient consent was requested and obtained prior to publication of clinical pictures. All clinical pictures are intraoral and do not include any identifying patient information.

### Virtual planning

**Figure 2:**
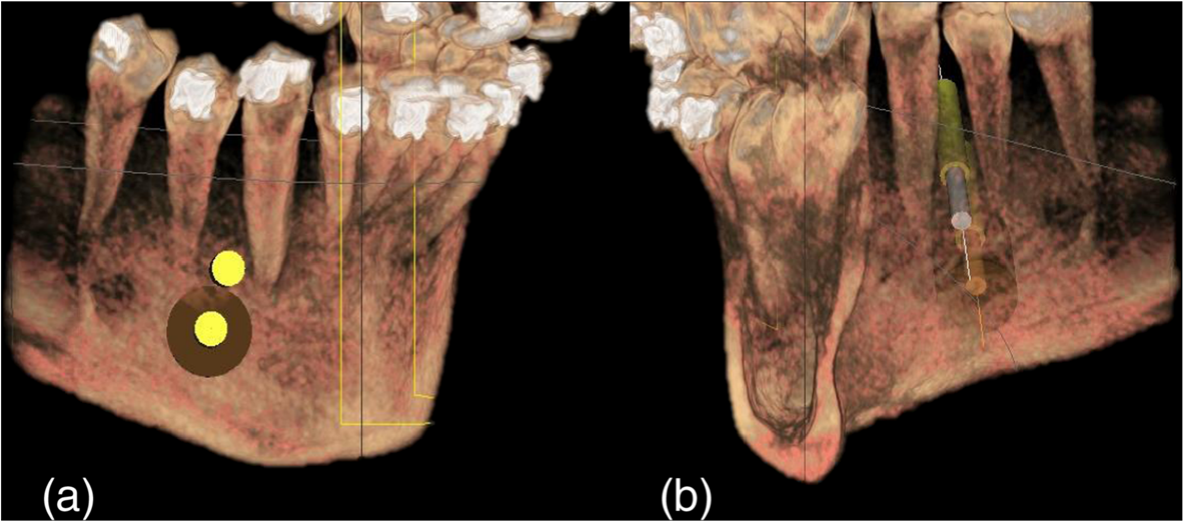
**Virtual planning of screw position.** (**a**) Buccal view, (**b**) Lingual view.

In order to perform all pre-surgical preparations, a cone-beam computed tomography (CT) scan of the patient is required. Dedicated software (Galaxis, Sirona, Bensheim, Germany) is used to construct a three-dimensional image of the scanned area and determine the preferred position of the mini-plate and the fixation screws (Figure 2a and b) [32]. The software provides the option of virtually placing the screws in the selected area of bone and allows the clinician to accurately visualize the desired result prior to surgery. In cases, for example, where space closure mechanics are designed to protract the posterior dentition, the mini-plate is placed in the area between the canine and the first premolar.

**Figure 3:**
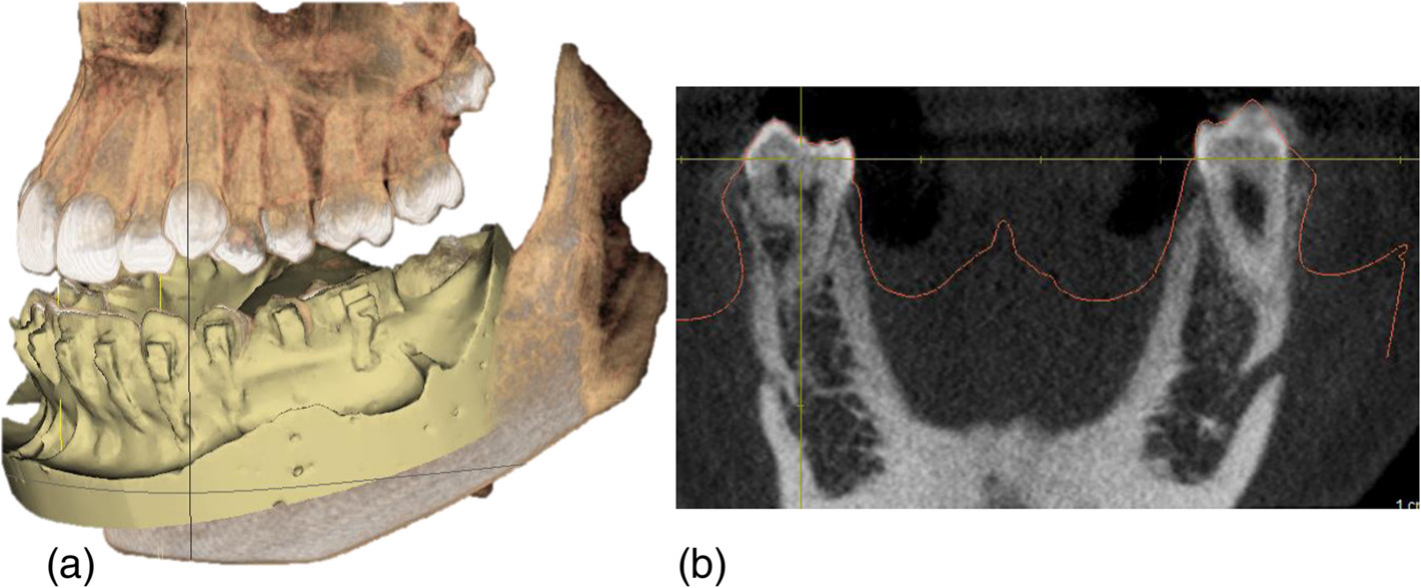
**Creating a virtual outline of the soft tissue contour on the CBCT image allows to precisely define the area where the connection bar of the mini-plate will penetrate the gingiva.** (**a**) Matching of the scanned plaster models to the CBCT image, (**b**) Creating a digital outline of the soft tissue contour.

In order to ensure precise placement of the plate through the mucogingival tissues, the software can also be used to match a scanned plaster model of the mandibular arch to the virtual image of the mandible and create an outline of the soft tissue contour (Figure 3a and b).

After virtual planning is complete, the CBCT dataset is converted from the initial DICOM (Digital Imaging and Communication in Medicine) format [33] to a Stereo-lithography (STL) format [34]. For this purpose, a DICOM imaging software (OsiriX®, Version 2.0.1, 64 Bit, Pixmeo, Bernex, Switzerland) for MacOS (Apple, Cupertino, Ca, USA) was used in the case presented in Figure 3. This conversion was undertaken, to input the CBCT dataset into a 3-D printer (Rasteder KFO - Spezial Labor, Rastede, Germany) and create a stereo-lithographic working model of the mandible.

The drill guide is fabricated by an external service provider (SICAT, Bensheim, Germany), also based on the virtual data.

### Adaptation of the bone plates on the stereo-lithographic model

Commercially available orthodontic anchorage plates (Promedia Medizintechnik, Siegen, Germany) are adapted to the stereo-lithographic model. The initial adaptation is simple, because their position is determined by the holes that were created with the help of the drill guide. Precise adaptation of the base and the connecting arm of the mini-plate to the contour of the anterior mandible are performed on the model with orthodontic pliers.

The plate is subsequently secured to the stereo-lithographic model using screws, and its adaptation is verified. The screws used in this stage are identical to the ones used during the actual surgical placement.

Finally, a transfer jig is fabricated with a light-cured tray material (Bredent, Senden, Germany), which is adapted to cover the incisal part of the mini-plate as well as the incisal edges of at least three teeth to key the position of the plate to those teeth when it is transferred to the patient’s mouth.

**Figure 4:**
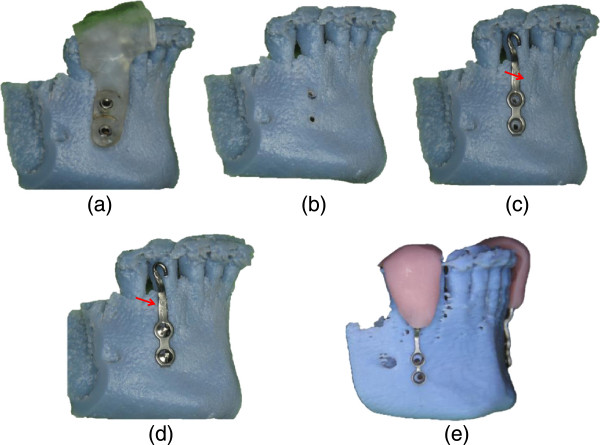
**Adaptation of the mini-plate. (a)** Placement of the drill guide, **(b)** Pilot holes made on the STL model, **(c)** Adaptation of the mini-plate, **(d)** Mini-plate secured with fixation screws, **(e)** Transfer-jig. (Note: Orthodontic pliers are used to make additional bends and insure good adaptation of the connection arm of the plate).

The complete process from plate adaptation to fabrication of the transfer jig on the stereolithographic model is presented in Figures 4 a-e.

### Surgical placement of the mini-plates

The surgical procedure is performed under local anesthesia. In most cases, a mental nerve block appears to be sufficient for this purpose.

**Figure 5:**
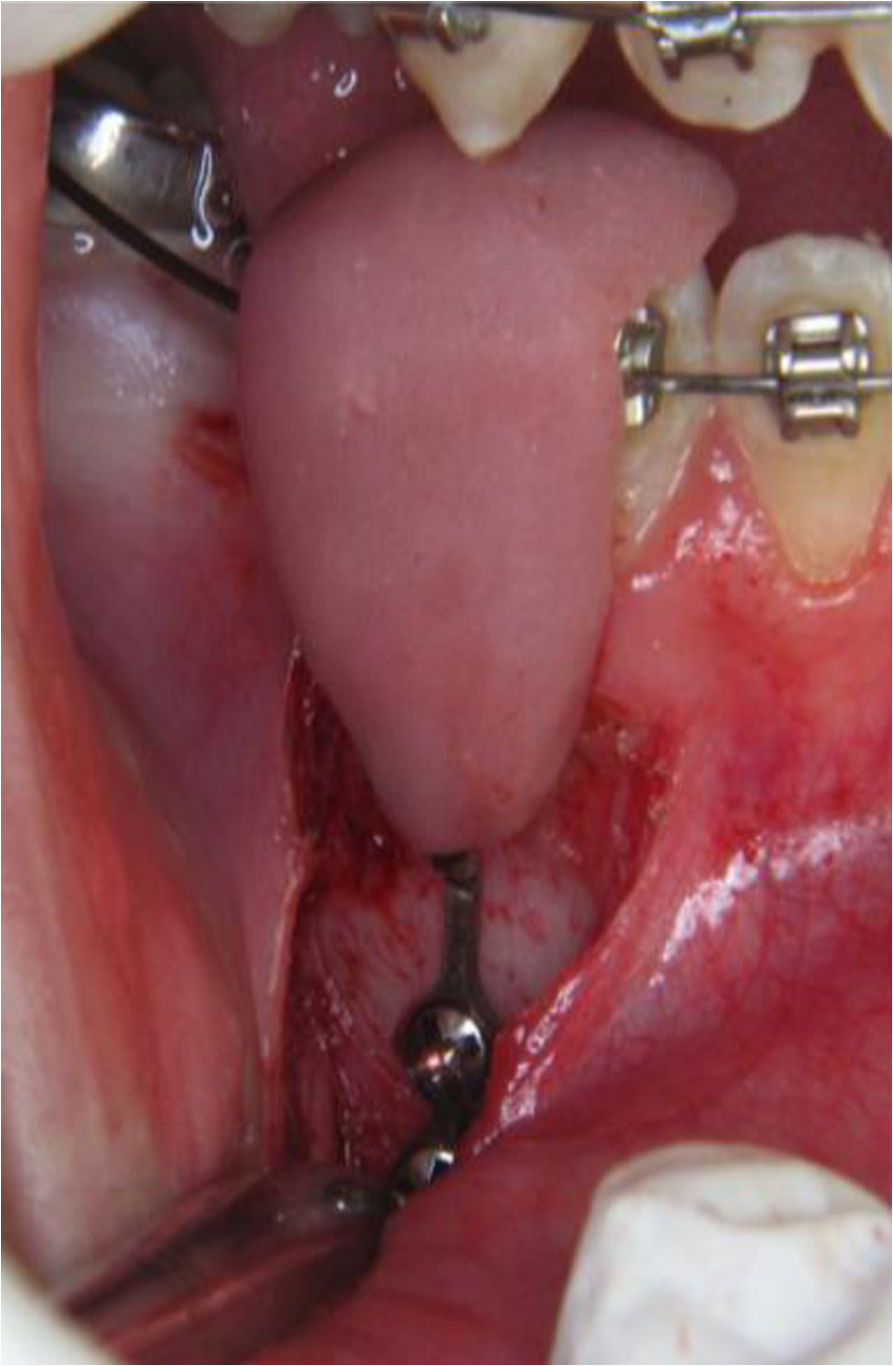
Intra-operative positioning of the transfer-jig and the retaining screws after elevation of the gingival tissue.

The type of surgical incision is based on the surgical site. In the case presented in Figure 5, an arcuate incision was made with the convex portion directed coronally. Regardless of the incision type, it needs to be ensured that the site where the connection bar of the mini-plates, including the orthodontic attachment, penetrates the gingival tissue is located in the attached gingiva [27].

Following the surgical incision, a full thickness flap is raised and the plate is transferred to the surgical site using the custom jig. The patient is asked to bite on the jig, in order to avoid minor movements or displacement. Once the correct position of the plate has been confirmed clinically, pilot holes are created and two 5 mm long and 2 mm in diameter titanium screws (Promedia Medizintechnik, Siegen, Germany) are inserted to secure the mini-plate on the mandibular bone.

**Figure 6:**
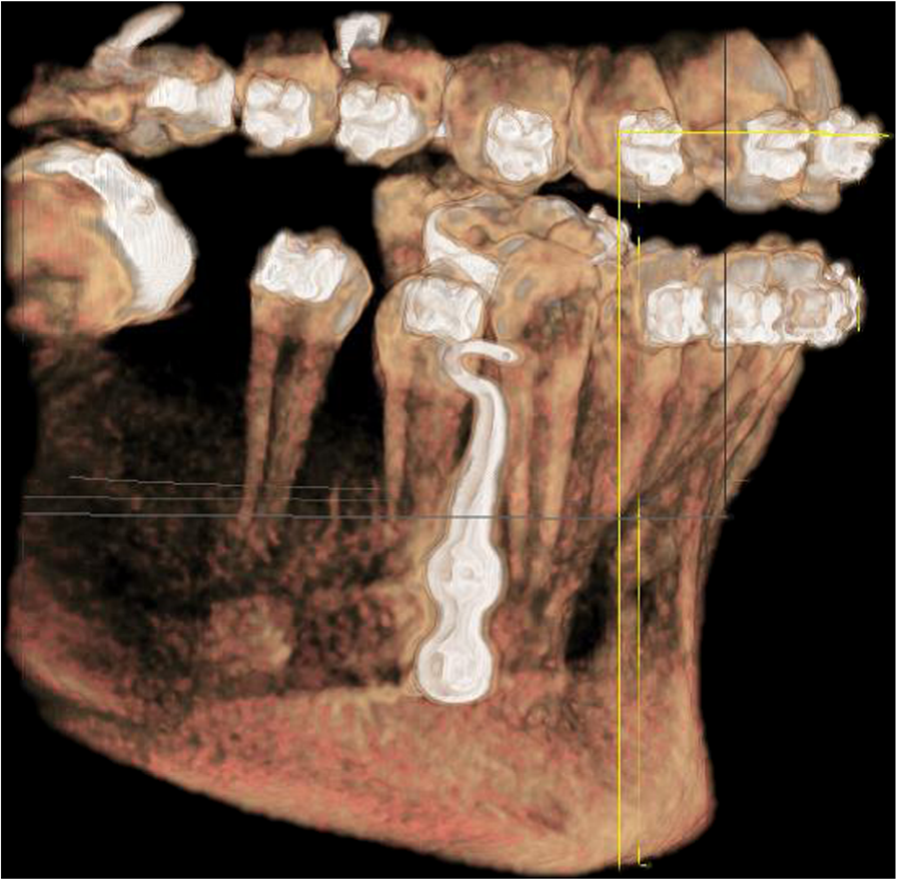
Post-operative limited CBCT scan.

Finally, the flap is sutured back into its initial position so that it covers the base of the mini-plate, but allows the connection bar to project coronally. Although it is not necessary, successful placement of the anchorage plates can be documented by means of a post-operative limited CBCT scan (Figure 6).

## Discussion

A novel technique is presented to precisely determine the desired final position of orthodontic mini-plates prior to surgery, and therby simplify as well as significantly reduce the time for surgical placement. A pre-operative CBCT image allows the clinician to thoroughly evaluate the bone around the insertion site in all three dimensions. Three-dimensional diagnostic imaging has been previously suggested to improve the outcome of implant placement by eliminating distortion errors associated with two-dimensional images and by reducing the risk of injury of adjacent structures [35,36].

Furthermore, virtual placement of the pilot holes and mini-plate on the CBCT image, using dedicated software, allows the clinician to precisely determine the final position of the plate during the surgery [32]. Several studies have proposed virtual treatment planning as a means for achieving higher surgical success rates by reducing intra-operative decision-making [35-38]. The duration of surgery can be crucial for its final outcome, since longer surgeries are associated to larger edemas and more post-operative pain [39].

The final step of pre-surgical preparation for the technique described here is the adaptation of the mini-plate on a stereolithographic model and the construction of a custom transfer jig. The fabrication of surgical guides on stereolithographic models for the placement of mini-implants has been described in the past [35,40], however, no similar technique has to our knowledge been reported for the placement of mini-plates. Pre-operative adaptation of the mini-plates on the model surface is the main advantage of the present method. It allows for maximum contact between the plate and the bony surface during the surgery and therefore significantly reduces the risk for infection and the possibility of mini-plate failure [30].

Despite its advantages, when our technique is compared to the use of mini-implants, it is indeed associated with more patient discomfort due to the need for a more extensive surgical procedure [41]. However, in certain areas of the maxilla and the mandible, the use of mini-implants is limited by the anatomy of the oral tissues or the quality of the bone [9]. In such cases, mini-plates can be used to provide effective skeletal anchorage.

Another potential concern of the present technique is the requirement for an initial CBCT. Radiation exposure for a sectional CBCT of the mandible ranges between 35 μSv – 113 μSv depending on the resolution [42,43]. The total effective dose of absorbed radiation from a mandibular CBCT is approximately 3.5 times larger compared to a panoramic x-ray [44] and 15 times larger compared to a periapical radiograph, depending on the area that is scanned [45]. These significant differences in radiation dosages need to be taken into serious consideration prior to applying the proposed method, especially in younger patients.

## Conclusions

A new technique is presented to improve the accuracy and potentially decrease the failure rate of surgical placement of orthodontic mini-plates. It is based on virtual treatment planning and accurate positioning of the plate with a custom made transfer jig. Despite the benefits of the technique, the need of an initial CBCT might be a limiting factor for using the method especially in younger patients due to increased radiation exposure.

## Competing interests

None of the authors have received reimbursements, fees, funding, or salary from an organization that may in any way gain or lose financially from the publication of this manuscript, either now or in the future.

This manuscript is not being funded by any organization.

None of the authors hold any stocks or shares in an organization that may in any way gain or lose financially from the publication of this manuscript.

The authors do not hold or are currently applying for any patents relating to the content of the manuscript nor have they received reimbursements, fees, funding, or salary from an organization that holds or has applied for patents relating to the content of the manuscript.

None of the authors of this manuscript have any financial or non-financial interests in the publication of this manuscript.

## Author’s contributions

JH conceived the project, gathered and processed the material presented (clinical pictures, electronic images etc.) and drafted the manuscript. GK edited the initial draft, critically revised it and created the final version of this manuscript. PG critically revised the manuscript and approved its final version prior to submission. BL conceived the idea for this manuscript, reviewed every step of the process and approved the final version of the manuscript. All authors read and approved the final manuscript.
